# Pulmonary recovery from COVID-19 in patients with metabolic diseases: a longitudinal prospective cohort study

**DOI:** 10.1038/s41598-023-29654-1

**Published:** 2023-02-14

**Authors:** Thomas Sonnweber, Philipp Grubwieser, Alex Pizzini, Anna Boehm, Sabina Sahanic, Anna Luger, Christoph Schwabl, Gerlig Widmann, Alexander Egger, Gregor Hoermann, Ewald Wöll, Bernhard Puchner, Susanne Kaser, Igor Theurl, Manfred Nairz, Piotr Tymoszuk, Günter Weiss, Michael Joannidis, Judith Löffler-Ragg, Ivan Tancevski

**Affiliations:** 1 Department of Internal Medicine II, Medical University of Innsbruck, Innsbruck, Austria; 2 Department of Radiology, Medical University of Innsbruck, Innsbruck, Austria; 3 Department of Internal Medicine I, Medical University of Innsbruck, Innsbruck, Austria; 4 Central Institute of Medical and Chemical Laboratory Diagnostics, University Hospital Innsbruck, Innsbruck, Austria; 5 MLL Munich Leukemia Laboratory, Munich, Germany; 6 Department of Internal Medicine, St. Vinzenz Hospital, Zams, Austria; 7 The Karl Landsteiner Institute, Reha Zentrum Münster, Münster, Austria; 8 Data Analytics As a Service Tirol, Innsbruck, Austria; 9 Division of Intensive Care and Emergency Medicine, Department of Internal Medicine I, Medical University of Innsbruck, Innsbruck, Austria

**Keywords:** Health care, Medical research

## Abstract

The severity of coronavirus disease 2019 (COVID-19) is related to the presence of comorbidities including metabolic diseases. We herein present data from the longitudinal prospective CovILD trial, and investigate the recovery from COVID-19 in individuals with dysglycemia and dyslipidemia. A total of 145 COVID-19 patients were prospectively followed and a comprehensive clinical, laboratory and imaging assessment was performed at 60, 100, 180, and 360 days after the onset of COVID-19. The severity of acute COVID-19 and outcome at early post-acute follow-up were significantly related to the presence of dysglycemia and dyslipidemia. Still, at long-term follow-up, metabolic disorders were not associated with an adverse pulmonary outcome, as reflected by a good recovery of structural lung abnormalities in both, patients with and without metabolic diseases. To conclude, dyslipidemia and dysglycemia are associated with a more severe course of acute COVID-19 as well as delayed early recovery but do not impair long-term pulmonary recovery.

## Introduction

Coronavirus disease 2019 (COVID-19) is caused by severe acute respiratory syndrome coronavirus type 2 (SARS-CoV-2) and remains a global health concern^1^. The course and outcome of COVID-19 depend on various risk factors, including age, gender, genetics, and comorbidities. Most prominently, metabolic diseases, including dysglycemia and dyslipidemia, are well-established risk factors for severe COVID-19^2–6^.

Studies from Chinese and Italian cohorts showed that the prevalence of diabetes in COVID-19 patients is not increased when compared to the general population, suggesting no increased risk of infection in patients with this comorbidity^7,8^. In contrast, diabetic patients suffering from COVID-19 are at higher risk for a severe or fatal course of disease including hospitalization, ICU admission, and need for mechanical ventilation^3,9–11^. Proposed pathogenetic links between COVID-19 severity and diabetes include poor glycemic control, aggravated systemic inflammation, altered immune response, and activation of the renin–angiotensin–aldosterone signaling pathway^9^.

Although several studies found dyslipidemia to be associated with increased COVID-19 mortality, a recent meta-analysis found no independent correlation, if effect estimates of dyslipidemia are adjusted for potentially confounding risk factors^12,13^. However, there is definitive evidence for the association between dyslipidemia and COVID-19 severity. A recent umbrella review (overview of systematic reviews) showed that a history of dyslipidemia is a risk factor for a severe course of acute COVID-19 (RR: 1.49), that acute SARS-CoV-2 infection alters lipid metabolism, and that both, HDL and LDL cholesterol concentrations, correlate inversely with severity of COVID-19^14^. Of note, rapid shifts in blood lipid levels have been reported in acute SARS-CoV-2 infection, which has consistently been observed with SIRS and bacterial sepsis, as well^15–17^. Several potential mechanisms explaining this altered lipid profile have been postulated, including decreased lipid synthesis during an excessive state of inflammation, liver dysfunction, capillary leakage, and a nutritional immune response^18^. A severe course of COVID-19 is characterized by a “SIRS-like” immunological response, showing a cytokine profile in plasma that does not differ from ARDS and sepsis^19^. Mechanistically, low levels of HDL cholesterol or impaired uptake of it into the adrenals may lead to reduced corticosteroid levels and an increased death rate in SIRS^20^. In line, low levels of HDL cholesterol were associated with severe outcomes in COVID-19, which was traced back to a pivotal role for cholesterol in the cellular entry of coronaviruses, including SARS and SARS-CoV-2^21–24^. Overall, hypocholesterolemia is a widely recognized prognosticator of poor outcomes in sepsis, attributable to its pleiotropic immunomodulatory effects^25^.

Besides low levels of HDL cholesterol, dyslipidemia is characterized by elevated serum triglyceride levels which were shown to associate with mortality in patients with COVID-19^26^. Mechanistically, our group previously demonstrated that hypertriglyceridemia may induce apoptosis in human macrophages and endothelial cells, which may crucially contribute to COVID-19 severity^27^.

Metabolic disease and obesity are associated with chronic low-grade inflammation^28,29^. Increased adipose tissue affects systemic metabolism through adaption in adipocytokines, including leptin and adiponectin. Leptin, which is mainly known for its crucial role in the control of energy homeostasis and is related to the quantity of fat mass, exerts immune-modulatory functions and alters glycemic control^30^. Adiponectin has a well-established anti-inflammatory and anti-oxidative function and is reduced in patients with metabolic disease and obesity^31,32^. Thus, considering the immune-modulatory effects of adipocytokines, it has been speculated that disturbances of adipocytokine expression in obesity and metabolic disease may directly contribute to a poor outcome of COVID-19. In this regard, a recent observational study linked a reduction of the adiponectin/leptin ratio, as found in patients with metabolic disease, to worse outcomes in COVID-19^33^. Alternatively, reduced adiponectin and increased leptin serum concentrations as surrogates of chronic inflammation may reflect metabolic disease which per se impacts the course of COVID-19. Moreover, SARS-CoV-2 was recently shown to infect adipocytes, in turn triggering adipose tissue dysfunction and insulin resistance^34,35^.

In summary, metabolic comorbidities appear to play an important role in the pathogenesis of acute COVID-19. Whether dysglycemia and dyslipidemia also influence the long term-term outcome after COVID-19 remains to be assessed.

We recently reported on the long-term sequelae and cardiopulmonary recovery of COVID-19 patients in the prospective observational CovILD (Development of Interstitial Lung Disease (ILD) in patients with COVID-19) study (NCT04416100), where we observed a high incidence of prediabetes, diabetes mellitus and dyslipidemia in critically ill COVID-19 patients, which was often undetected before SARS-CoV-2 infection^36,37^. We herein aim to dissect the impact of dysglycemia and dyslipidemia on the long-term recovery from COVID-19 until 360 days after disease onset.

## Materials and methods

During the outbreak of COVID-19 in Austria with its first major hot spot in the Alpine region of Tyrol in early 2020, a prospective, multicentre, observational follow-up study was initiated at the Department of Internal Medicine II, at the Medical University of Innsbruck CovILD trial^36^. The study includes hospitalized COVID-19 patients, as well as COVID-19 outpatients. The diagnosis was confirmed by typical clinical presentation along with a positive RT-PCR SARS-CoV-2 test result obtained from a nasopharyngeal or oropharyngeal swab. After enrollment, patients were offered a comprehensive medical assessment at four follow-up visits (visit 1 = 60 days, visit 2 = 100 days, visit 3 = 180 days, and visit 4 = 360 days after COVID-19 onset, respectively). Acute COVID-19 disease severity was graded according to the need for medical treatment: mild: outward treatment, moderate: hospital treatment without oxygen supplementation, severe: hospitalization with oxygen supplementation or intensive care unit treatment. Notably, during the study recruitment period, the Tyrolian healthcare system was never overloaded, thus, all patients received supportive care according to the standard of care at the trial site hospitals and no selection bias due to triage methods was apparent. Thus, only patients with mild clinical symptoms (without significant respiratory or circulatory impairment) during acute COVID-19 received outward treatment and were categorized as “mild”.

Data on clinical characteristics, laboratory testing, lung function and low-dose computed tomography (CT) were evaluated at each time point. Pulmonary CT images were analyzed for the presence and distribution of ground-glass opacities (GGO), consolidations, bronchial dilation, and reticulations. A CT severity score, generated by three independent radiologists, and an automated software-based pneumonia grading, using Syngo.via CT Pneumonia Analysis prototype software (Siemens Healthineers, Erlangen, Germany), were used to grade the level of pulmonary impairment at all follow-ups, as previously reported^36,38^. Analysis of the following parameters was conducted at the Central Institute of Medical and Chemical Laboratory Diagnostics of the University Hospital Innsbruck according to the manufacturers’ procedures: HbA1c (Tosoh G8); total cholesterol, triglycerides, C-reactive protein (CRP), interleukin-6 (IL-6), ferritin (Roche Cobas 8000 analyzer); adiponectin (BEP2000 using reagent from Biovendor); leptin, hepcidin (BEP 2000 using reagent from DRG Instruments); D-dimer (Siemens BCS-XP instrument using the Siemens D-Dimer Innovance reagent)^36,39^. Dysglycemia was defined by the presence of prediabetes (HbA1c ≥ 5.7%) or diabetes (HbA1c ≥ 6.5%) assessed at early post-acute follow-up, respectively. Dyslipidemia was defined when both, hypertriglyceridemia (serum triglycerides > 200 mg/dL) and reduced HDL-C (HDL-C < 40 mg/dL for males and < 50 mg/dL for females) were present at follow-up, as this combination is most likely related to inflammation triggered insulin resistance^40^.

Statistical analysis and data visualization were performed using the GraphPad Prism software version 8.0 (GraphPad Software, San Diego, CA, USA), statistical analysis software package (IBM SPSS Statistics version 27.0; IBM, Armonk, NY, USA) and R version 4.2.0. A detailed description of the statistical analysis is provided in the Supplementary Methods section.

### Institutional review board statement

The study was conducted according to the guidelines of the Declaration of Helsinki and approved by the Institutional Review Board of the Medical University of Innsbruck (approval number: 1103/2020), and the study is registered at ClinicalTrials.gov (registration number: NCT04416100).

### Informed consent statement

Written informed consent was obtained from all subjects involved in the study.

## Results

### Patients’ baseline characteristics

A total of 145 patients were included in the study, with 138 at 100 days, 119 at 180 days, and 92 at 360 days follow-up available for analysis. Patients with mild to moderate COVID-19 demonstrated a higher dropout rate (45%) compared to patients with severe to critical COVID-19 (30%), resulting in a moderate selection bias for individuals who suffered from more severe COVID-19 at later follow-up time points. Dropout was exclusively due to patients’ unwillingness to continue the study, whereas no patient died during follow-up. According to the need for medical treatment, 24% of patients had mild (N = 34), 26% moderate (N = 38), and 50% severe (N = 73) acute COVID-19. The baseline characteristics of the study cohort are presented in Table 1. Symptomatic presentation during acute COVID-19 included dyspnea (68%), cough (70%), fever (73%), thoracic pain (54%), hyposmia/anosmia (43%) and diarrhoea/vomiting (41%).

**Table 1:** Baseline characteristics of the study cohort (N = 145).

Patient characteristics	
Mean age (years (SD))	57.3 (14.3)
Gender (N female (%))	63 (43.4)
BMI and obesity grading	
Mean BMI (kg/m^2^ (SD))	26.4 (4.7)
BMI < 25 kg/m^2^ (N (%))	57 (39.3)
BMI 25–30 kg/m^2^ (N (%))	59 (40.7)
BMI 30–35 kg/m^2^ (N (%))	21 (14.5)
BMI 35–40 kg/m^2^ (N (%))	6 (4.1)
BMI ≥ 40 kg/m^2^ (N (%))	2 (1.4)
Comorbidities	N (%)
No comorbidities	33 (22.8)
≥ 3 comorbidities	74 (51.0)
Metabolic diseases	63 (43.4)
Diabetes mellitus type 1	0 (0.0)
Diabetes mellitus type 2	24 (16.6)
Hypercholesterolemia	27 (18.6)
Hypertriglyceridemia	2 (1.4)
Cardiovascular disease	58 (40.0)
Arterial hypertension	44 (30.3)
Pulmonary disease	27 (18.6)
Chronic kidney disease	10 (6.9)
Chronic liver disease	8 (5.5)
Gastrointestinal disease	20 (13.8)
Malignancy	17 (11.7)
Immune suppression	9 (6.2)

### Metabolic diseases in the CovILD study cohort

**Figure 1:**
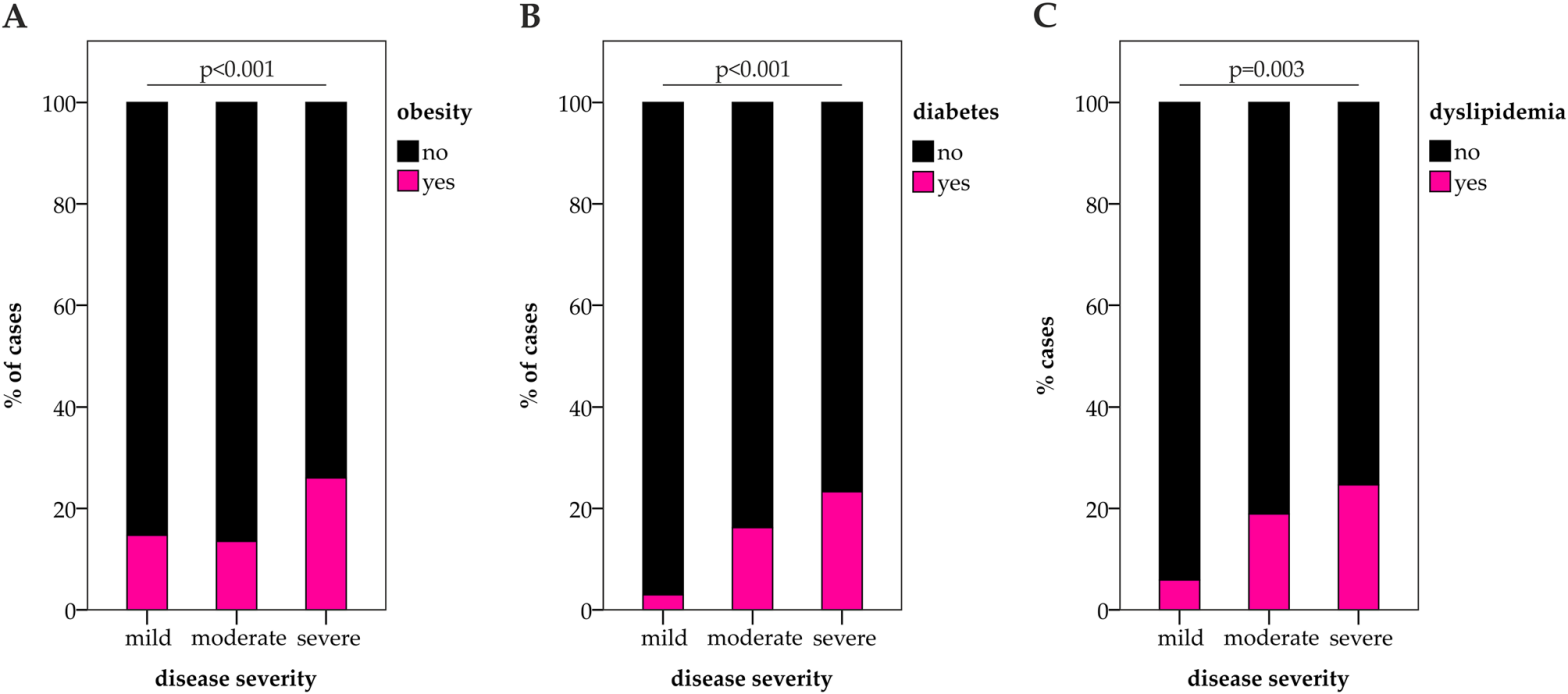
Preexisting metabolic comorbidities are related to the course of acute COVID-19. Bars depict the prevalence of obesity (**A**), diabetes mellitus (**B**), and dyslipidemia (**C**) before COVID-19 onset in patients with mild, moderate and severe acute COVID-19. N_total_ = 145, N_mild_ = 34, N_moderate_ = 38, N_severe_ = 73. *P*-values are reported according to Chi-Square tests.

According to medical history, metabolic disorders were highly frequent in the CovILD study cohort and 43% of the patients presented with metabolic comorbidities, including overweight and obesity (N = 88, 61%), diabetes mellitus (N = 24, 17%), and dyslipidemia (N = 27, 19%). Of note, these preexisting metabolic diseases were associated with the course of acute COVID-19, as patients with obesity, dysglycemia, and dyslipidemia more frequently developed severe acute COVID-19 as compared to those without these comorbidities (Fig. 1). Accordingly, hospitalized patients with preexisting metabolic diseases such as diabetes mellitus, obesity or dyslipidemia demonstrated a more severe structural lung involvement during acute COVID-19 as compared to individuals without preexisting metabolic disorders (mean CT severity score with metabolic disease: 16.0 ± 2.9 pts; mean CT severity score without metabolic disease: 11.8 ± 5.6, *P* = 0.033, N = 28).

**Figure 2:**
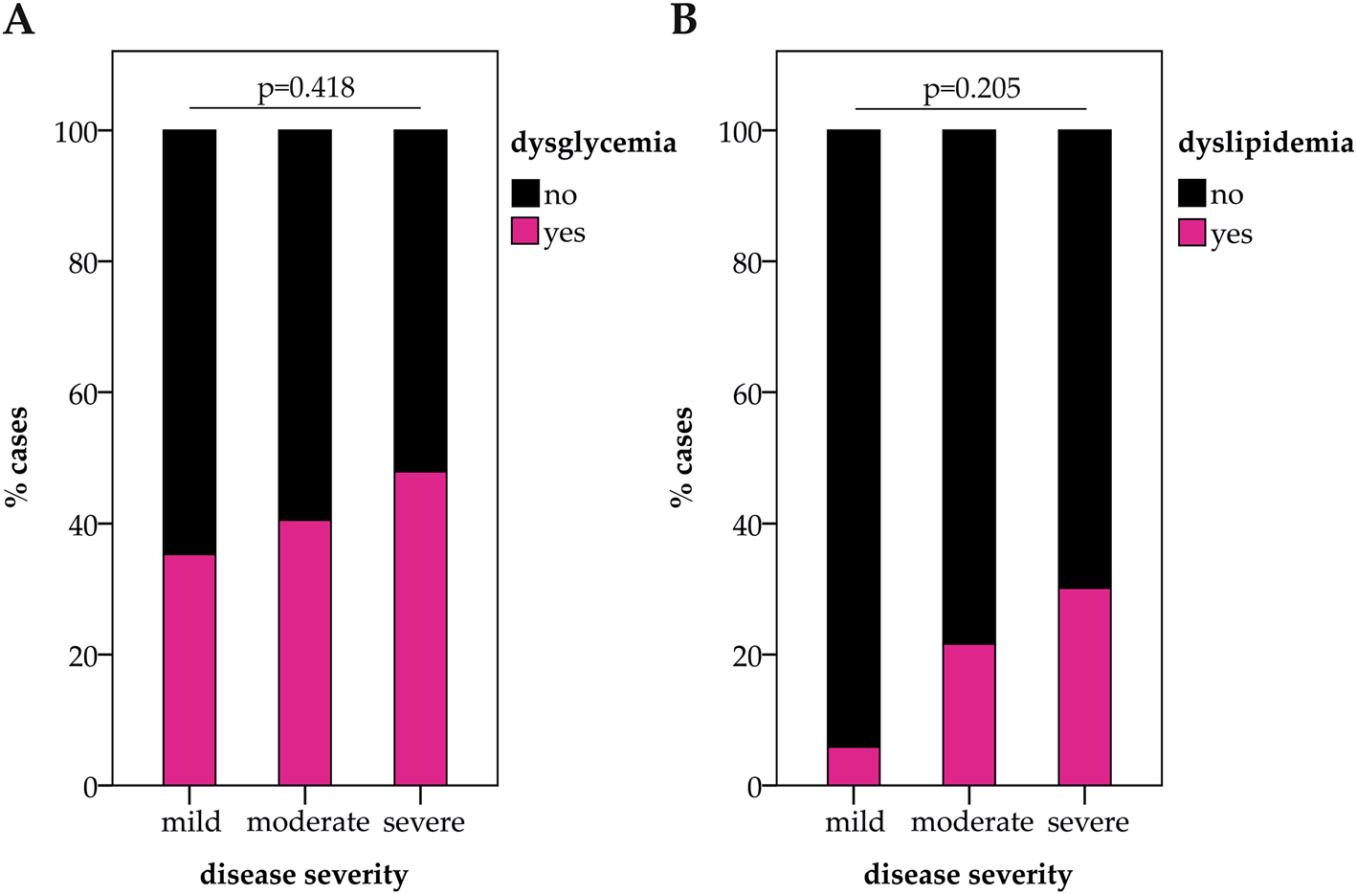
Dysglycemia and dyslipidemia at early post-acute follow-up after COVID-19. Patients were prospectively followed after acute COVID-19 and the presence of dysglycemia (**A**) and dyslipidemia (**B**) were assessed at 60 days post-COVID-19 follow-up. Bars depict the prevalence of dysglycemia and dyslipidemia in patients with mild, moderate and severe acute COVID-19. N_total_ = 145, N_dysglycemia_ = 62, N_dyslipidemia_ = 32. *P*-values are reported according to Chi-Square tests.

At early post-acute follow-up (60 days after the onset of COVID-19) a high portion of patients demonstrated dysglycemia (N = 62, 43%), defined by an HbA1c ≥ 5.7%, and dyslipidemia with low HDL-C and high triglyceride levels (N = 32, 22%). At this early post-acute follow-up, dyslipidemia and dysglycemia still tended to be of a higher prevalence in the patients with more severe acute COVID-19 but at this time-point differences were not significant anymore (Fig. 2).

**Figure 3:**
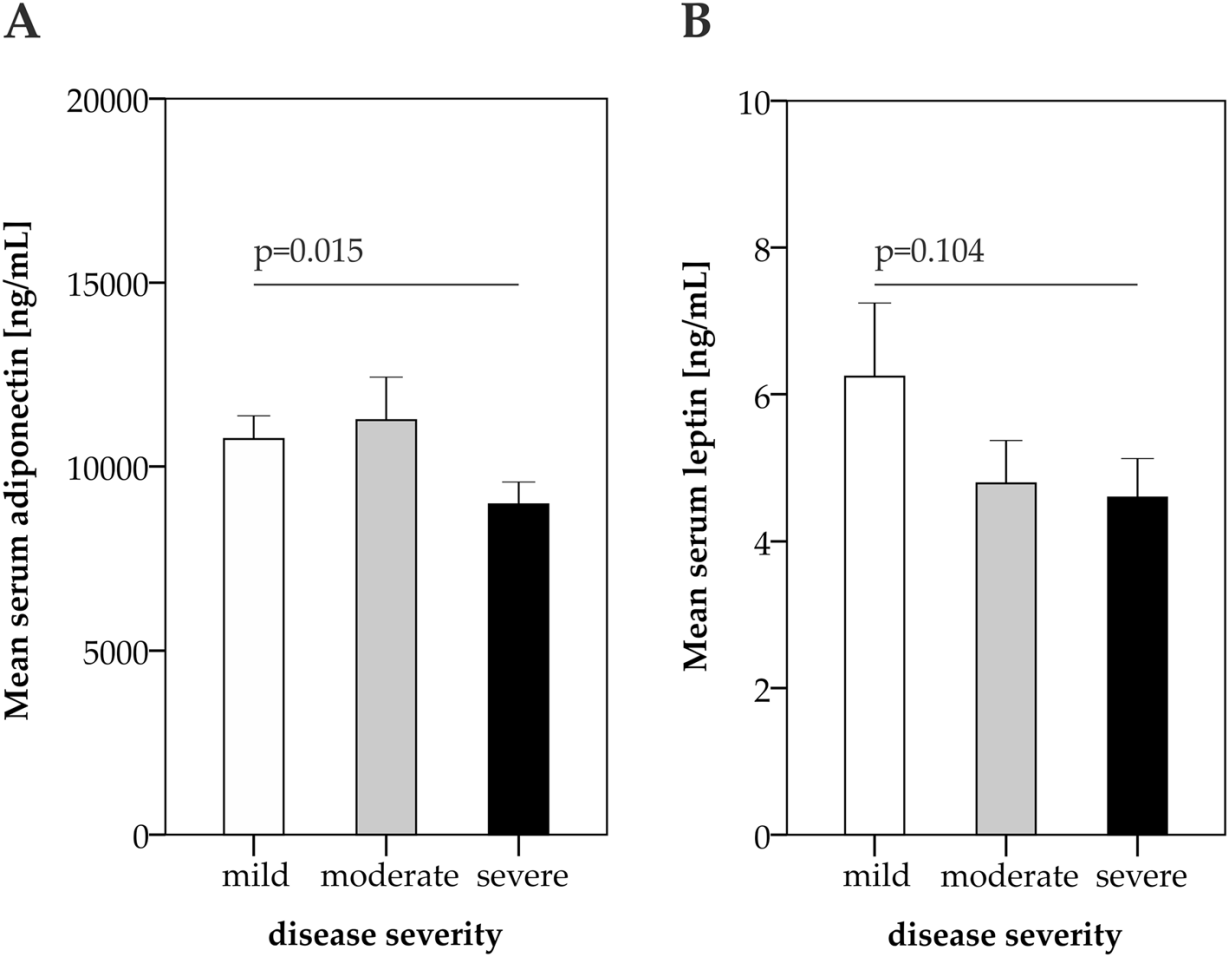
Adipocytokine concentrations at early post-acute follow-up are related to acute COVID-19 severity. Adiponectin (**A**) and leptin (**B**) serum concentrations at 60 days post-COVID-19 follow-up. Patient subgroups are shown according to the acute COVID-19 severity. Error bars depict one standard error. N_mild_ = 34, N_moderate_ = 38, N_severe_ = 73. *P*-values were determined using the Kruskal–Wallis test.

Interestingly, adiponectin serum concentrations were significantly lower in individuals who suffered from more severe acute COVID-19 (Fig. 3A). In contrast, leptin levels were not significantly different between clinical severity categories, although they also tended to be lower in patients with moderate and severe acute COVID-19 as compared to individuals who suffered from mild acute COVID-19 (Fig. 3B). At early post-acute follow-up, correlation analysis revealed that the severity of structural lung abnormalities was associated with age, inflammatory biomarkers, HDL-C and leptin blood levels (Table 2). Still, adipocytokines were more strongly associated with BMI than with the severity of structural lung impairment at early post-acute follow-up (serum leptin:BMI ρ = 0.467, *P* < 0.001, serum adiponectin:BMI ρ = 0.337, *P* < 0.001). Notably, 73% of patients reported a weight loss due to acute COVID-19 (5.7 ± 5.6 kg, mean ± SD), and the reduction of body weight was related to the severity of the acute disease (mean weight loss in kg ± SD: mild acute COVID-19: 2.2 ± 3.0 kg, moderate acute COVID-19: 3.4 ± 3.0 kg, severe acute COVID-19: 8.6 ± 6.0 kg).

**Table 2:** Correlation of biomarkers with structural lung impairment at early post-acute follow-up.

Baseline Characteristics	Spearman ρ	*P*-value
Body-mass index (BMI)	0.180	0.884
Age	0.521	< 0.001
Inflammatory Biomarker		
C-reactive protein	0.359	< 0.001
IL-6	0.425	< 0.001
Serum ferrtin	0.236	0.008
D-dimer	0.368	< 0.001
Blood lipids		
Triglycerides	0.106	0.235
HDL-C	− 0.209	0.019
Adipocytokines		
Serum adiponectin	− 0.035	0.694
Serum leptin	− 0.217	0.015

N = 145. Biomarkers were correlated with the severity of structural lung impairment as assessed by Syngo.via CT Pneumonia Analysis software at 60 days post-COVID follow-up.

### Thrombo-inflammatory serum biomarkers in patients with dysglycemia and dyslipidemia during the post-COVID-19 follow-up

**Figure 4:**
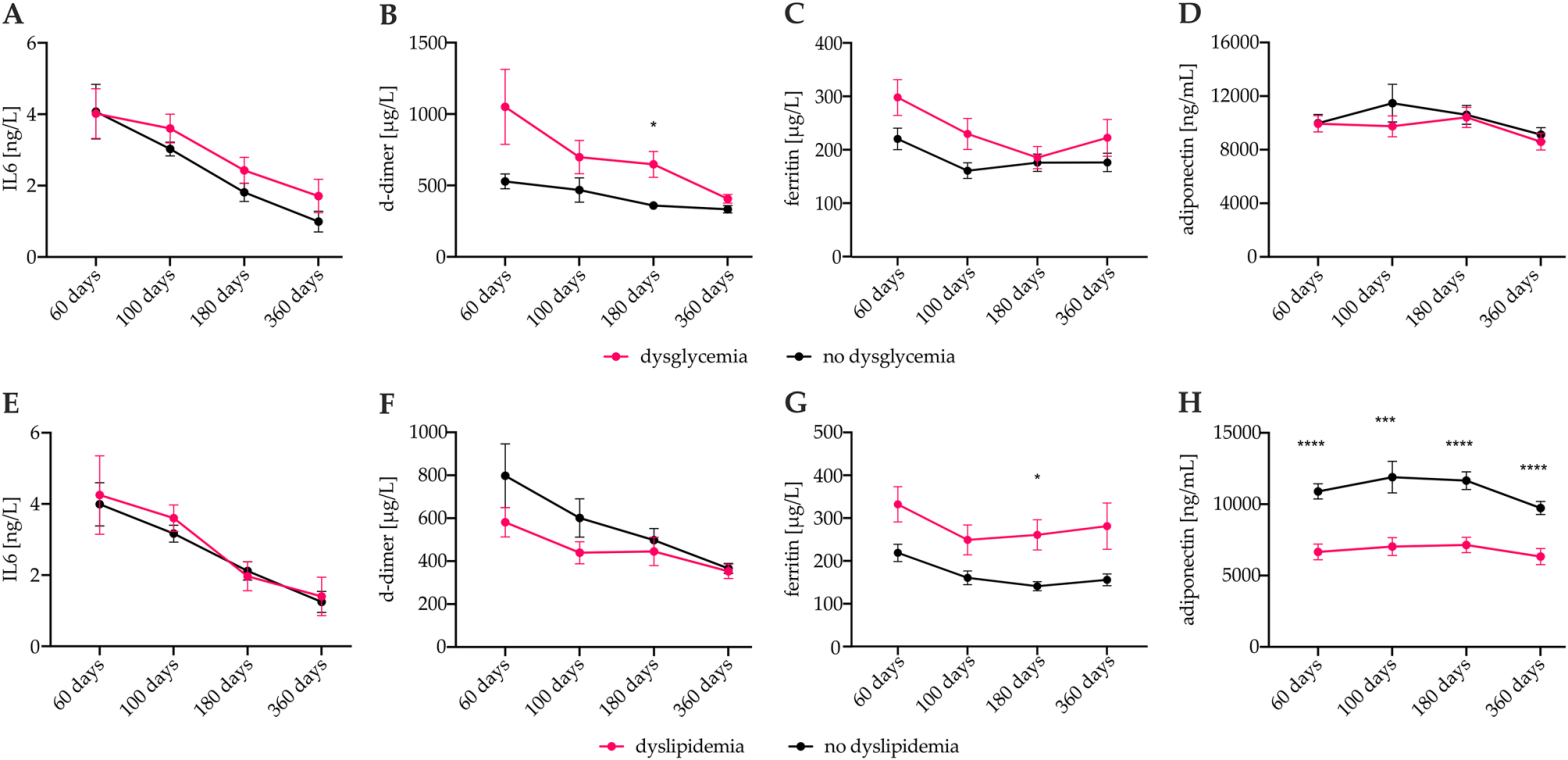
Serum markers of thrombo-inflammation in patients with dysglycemia and dyslipidemia at post-COVID-19 follow-up. COVID-19 patients were prospectively followed after acute COVID-19 and concentration of IL-6 (**A**, **E**), d-dimer (**B**, **F**), ferritin (**C**, **G**), and adiponectin (**D**, **H**) were prospectively assessed at 60, 100, 180, and 360 days after the onset of COVID-19. Subgroups are presented according to the presence of dysglycemia (A-D) or dyslipidemia (E–H), and statistical differences between these subgroups at each time point are indicated according to Mann Whitney-U tests *P < 0.05 and ***P < 0.001. Points depict means, error bars indicate standard error (SE). N_60days_ = 145, N_100days_ = 138, N_180days_ = 119, N_360days_ = 92.

To assess the impact of pro-inflammatory biomarkers in patients suffering from dysglycemia and dyslipidemia as compared to individuals without metabolic diseases, we longitudinally monitored the concentration of pro-inflammatory and pro-thrombotic biomarkers such as interleukin-6 (IL-6), C-reactive protein (CRP), serum ferritin, and d-dimer as well as adiponectin at 60, 100, 180 and 360 days after the onset of COVID-19 (Fig. 4). Patients with metabolic diseases tended to have higher pro-inflammatory biomarkers and markers of systemic inflammation correlated with adipocytokine expression and blood lipid concentrations (Supplementary Fig. 1). Of interest, we also found significantly lower adiponectin levels in patients with dyslipidemia. Notably, a significant drop in the investigated pro-inflammatory and pro-thrombotic mediators was found in both, patients with and without metabolic disease.

### Pulmonary recovery after COVID-19 in patients with and without metabolic diseases

**Figure 5:**
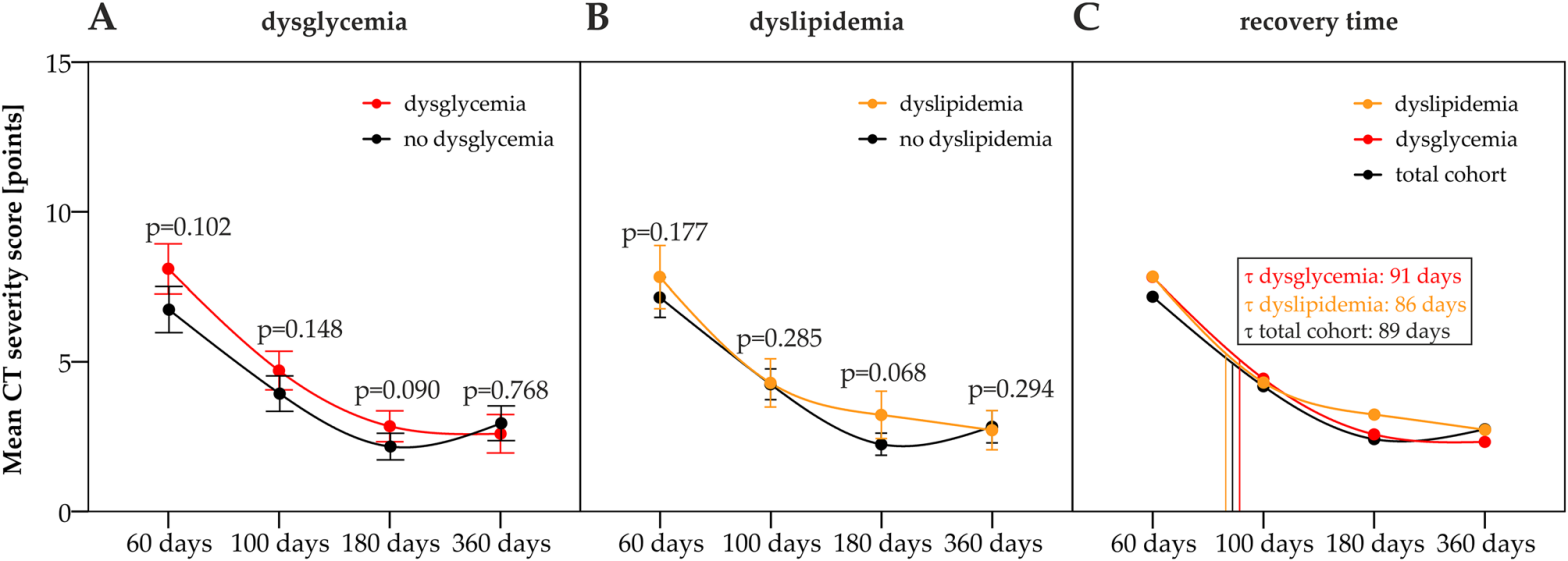
Pulmonary recovery after COVID-19 in patients with dysglycemia or dyslipidemia. Structural pulmonary impairment was assessed with computed tomography (CT) at four post-COVID-19 follow-ups. The severity of structural lung abnormalities was graded from 0 to 25 points (the higher the more severe) as described in the methods section. Time-dependent structural lung recovery according to the presence/absence of dysglycemia (**A**) or dyslipidemia (**B**) are presented. (**C**) Structural lung recovery time resulting in a 50 percent (τ) reduction of CT lung impairment as compared to the early post-acute follow-up in individuals with dysglycemia, dyslipidemia, and the total cohort are shown, whereas we found no significant differences in lung recovery time between the investigated subgroups. Mann–Whitney-U test (**A**, **B**) and Kruskal–Wallis test (**C**) were performed to analyze statistical differences between subgroups for each time point. Points indicate means, error bars depict standard error (SE). N_60days_ = 145, N_100days_ = 138, N_180days_ = 119, N_360days_ = 92.

Finally, we shed light on the structural pulmonary recovery, assessed with computed tomography (CT) at 60, 100, 180, and 360 days after COVID-19 onset in patients with or without metabolic disease. As previously mentioned, patients with metabolic disorders demonstrated more severe acute disease, thus at early post-acute follow-up, individuals with dyslipidemia or dysglycemia showed a non-significant trend towards more severe structural lung abnormalities as compared to individuals without metabolic diseases (Fig. 5A,B and Supplementary Fig. 2). At longitudinal follow-up both, patients with and without metabolic diseases demonstrated a pronounced lung recovery, and no significant difference in structural lung abnormalities was found when comparing patients with or without metabolic disease at the 100, 180, and 360 days post-COVID-19 follow-up. Moreover, the structural lung recovery time in patients with dyslipidemia or dysglycemia did not significantly differ from those without metabolic diseases (Fig. 5C).

In line with the imaging follow-up, we also performed lung function testing. As expected by the inclusion of predominately severe acute COVID-19 patients included in the CovILD cohort, a high portion of individuals demonstrated a lung function impairment at post-COVID-19 follow-up. In detail, abnormal lung function reflected by a reduction of the forced vital capacity (FVC), forced expiratory volume in one second (FEV1), total lung capacity (TLC) and/or diffusing capacity for carbon monoxide (TLcO), was found in 37% at the 60 days post-COVID-19 follow-up and 28% at the 180 days post-COVID-19 follow-up, whereas an impaired TLcO was the most frequent finding. Notably, the frequency of lung function impairment was not significantly related to the presence of dysglycemia and/or dyslipidemia at early or late post-COVID-19 follow-up.

**Figure 6:**
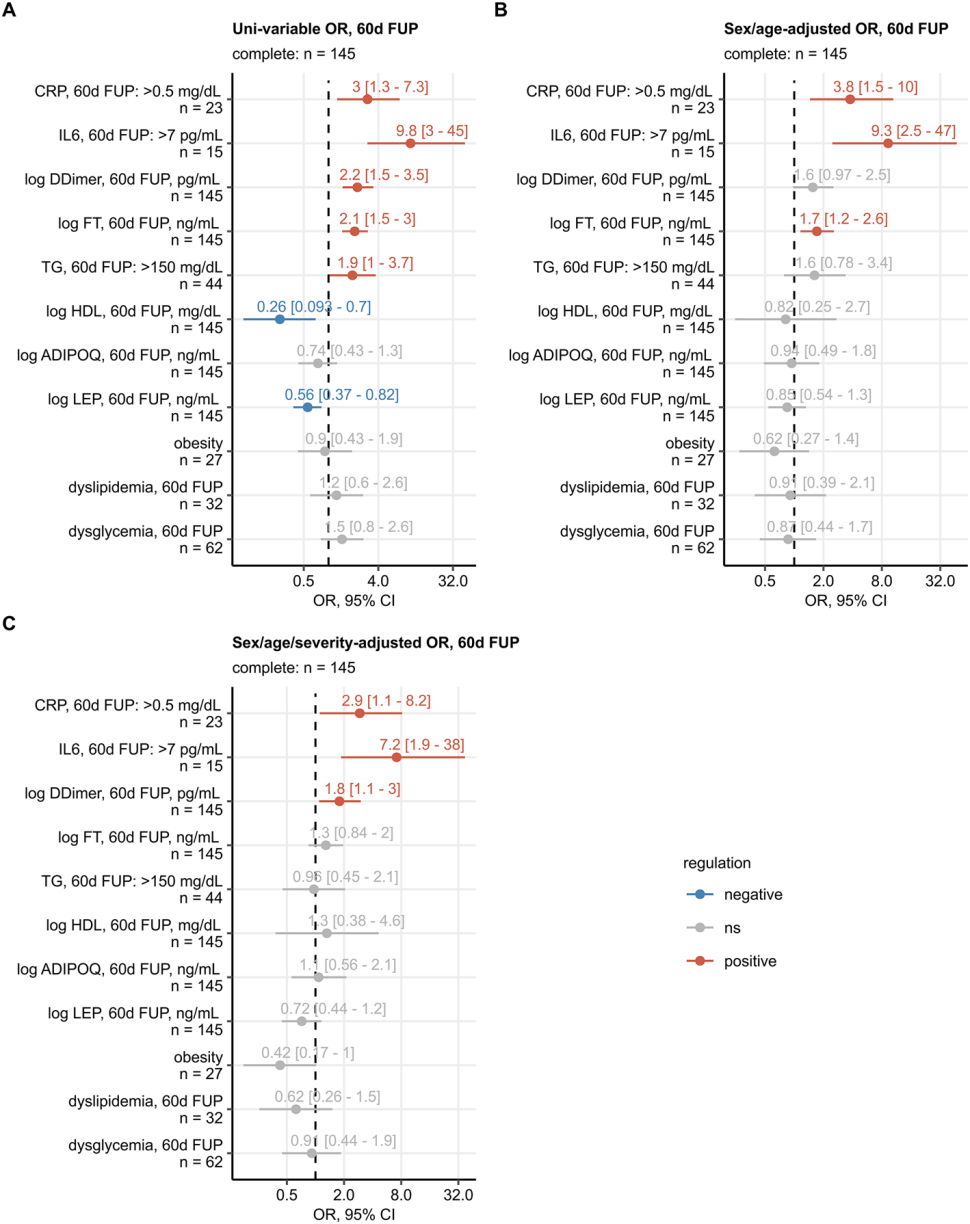
Logistic ordinal modeling of chest CT abnormality severity at the 60-day follow-up as a function of inflammatory and metabolic parameters. Chest CT abnormalities were classified as none (CT severity score [CTSS]: 0), mild (CTSS: 1–5), moderate (CTSS: 6–10) and severe (CTSS: $\ge$ 11). Effects of inflammatory (C-reactive protein [CRP], interleukin 6 [IL6], d-dimer [DDimer], ferritin [FT]), metabolic biomarkers (triglycerides [TG], high-density lipoprotein [HDL], adiponectin [ADIPOQ], leptin [LEP]) and metabolic disorders (obesity, dyslipidemia and dysglycemia) on chest CT abnormality severity at the 60-day follow-up were assessed by ordinal logistic regression. Odds ratio (OR) with 95% confidence intervals (CI) for the explanatory variables in univariable models (**A**), models adjusted for age class and sex (**B**) and models adjusted for age class, sex and acute COVID-19 severity (**C**) are shown in Forest plots. Point colour codes for significance and model estimate sign. Points are labeled with OR and 95% CI values.

Finally, we performed logistic ordinal modeling to assess the severity of chest CT lung abnormality as a function of inflammatory and metabolic parameters (Fig. 6, Supplementary Figs. 3, 4 and 5, and Supplementary Tables 1, 2, 3 and 4). This analysis revealed that persisting inflammatory parameters, such as CRP, IL-6, d-dimer and ferritin as well as acute COVID-19 severity predicted the risk for persistence of structural lung abnormalities at follow-up, whereas dyslipidemia and dysglycemia did not (Fig. 6). Accordingly, longitudinal evaluation with multi-parameter logistic ordinal modeling of CT lung abnormality severity as a function of age, sex, acute COVID-19 severity, and inflammatory and metabolic biomarkers indicate higher age, male sex and acute COVID-19 severity as risk factors for persisting structural lung impairment, whereas dyslipidemia or dysglycemia were not significantly associated with an impaired structural lung recovery (Supplementary Figs. 3, 4 and 5).

## Discussion

Dysglycemia and dyslipidemia are among the most frequent comorbidities found in COVID-19 patients^41–43^. Interestingly, current data suggest that dysglycemia and dyslipidemia do not increase the risk for SARS-CoV-2 infection, but are related to an adverse outcome in COVID-19, as reflected by an increased rate of intensive care unit (ICU) admission and higher mortality of COVID-19 patients with metabolic comorbidities including obesity and diabetes mellitus^12,42,44–47^. Notably, not only the diagnosis of diabetes mellitus but also the achievement of glycemic control per se were related to the outcome of severe COVID-19^48^.

Pathomechanisms explaining the relation of dyslipidemia and dysglycemia with a deteriorated outcome of acute COVID-19 have not been fully elucidated yet. Still, the association of metabolic disorders with chronic inflammation, impairment of immune response, endothelial dysfunction, and coagulopathy have been suggested as potential causes^49–51^. Additionally, acute hyperglycemia induces the expression of the angiotensin II converting enzyme (ACE2), which serves as a coronavirus surface protein receptor^52^. At present, however, it remains unclear to which extent hyperglycemia may facilitate cellular entry of SARS-CoV-2 into lung epithelial cells during acute disease. Infection with SARS-CoV-2 but also specific treatments including corticosteroids may aggravate pre-existing dyslipidemia or dysglycemia, or even induce a new onset of metabolic diseases, as suggested by data describing a high prevalence of inadequate glucose control in hospitalized COVID-19 patients^5,6,34,35,47,48,53,54^.

Dysglycemia and dyslipidemia are not only associated with a more severe course of the acute disease but may also contribute to impaired recovery from COVID-19. Still, to the best of our knowledge, there is currently no data available for prospectively evaluating the impact of metabolic diseases on the recovery from COVID-19. Thus, we herein shed light on this topic by investigating the outcome of patients with dysglycemia and/or dyslipidemia in the Austrian CovILD study cohort.

In line with various other studies and meta-analyses, the CovILD study demonstrates that both, dysglycemia and dyslipidemia, are associated with a more severe course of acute COVID-19. The latter also resulted in a trend towards more severe structural pulmonary involvement, as assessed with CT at early post-acute COVID-19 follow-up (60 days after disease onset). Notably, as this study is observational, a causative link between the presence of metabolic alterations and impaired early recovery cannot be established. At the early post-acute follow-up, patients with dysglycemia and dyslipidemia presented with significantly higher concentrations of thrombo-inflammatory biomarkers, including d-dimer and ferritin. These results support the previously mentioned theory that dyslipidemia and dysglycemia are associated with an impaired immune response, a more sustained inflammatory activity as a consequence of impaired viral control, and a subsequent prolonged inflammatory state in COVID-19^52^. Still, at long-term follow-up these differences dissolved, as serum concentrations of inflammatory biomarkers and the severity of structural lung abnormalities did not significantly differ according to the presence of dysglycemia or dyslipidemia at the 180- and 360 days post-COVID-19 follow-up evaluations. Thus, the trend towards more severe structural lung abnormalities and the increased markers of thrombo-inflammation, observed at the early post-acute COVID-19 follow-up, are more likely related to acute disease severity and may not reflect an impaired recovery or persistent/chronic immunological dysbalance in patients with dysglycemia or dyslipidemia. The latter is also supported by the observation that the presence of dysglycemia or dyslipidemia was not associated with a prolonged structural lung recovery time. In line with these results, lung function impairment at long-term follow-up was not significantly related to the presence of metabolic disorders such as dysglycemia or dyslipidemia. Still, it has to be noted, that this finding may not be true for patients with severe metabolic disorders, such as patients with vertebral fractures due to severe metabolic disease, who were reported to have impaired lung function recovery post-COVID-19^55^.

We also shed light on the expression of adipocytokines including adiponectin, which is known to exert immunomodulatory functions potentially contributing to the course of COVID-19^30–32^. Interestingly, the expression of adiponectin was related to acute COVID-19 severity, and patients with severe COVID-19 demonstrated decreased serum levels of adiponectin at the early post-acute follow-up when compared to individuals with mild or moderate acute disease. Reduced serum adiponectin concentrations are typically associated with increased markers of inflammation in infectious diseases, and respiratory failure in particular^56,57^. Theoretically, a lack of adiponectin, which mainly exerts anti-inflammatory functions, may contribute to the resolution of COVID-19, still, the herein presented study is solely observational, and thus, does not evaluate this potential pathomechanism in detail. Additionally, given the multifactorial regulation of adipocytokines, it is likely that various inputs simultaneously alter adipocytokine expression in patients suffering from COVID-19, including disease-related inflammation and metabolic changes related to weight loss. The latter may present an important factor for the herein presented data, as weight loss was significantly higher in patients with more severe COVID-19, and changes in fat mass quantity are a major regulator of adipocytokine expression^30–32^.

Mild hyperglycemia and new-onset diabetes mellitus have been repeatedly observed in patients affected by COVID-19 and were found to associate with worse outcomes. Mechanistically, dysglycemia and diabetes may be traced back to stress hyperglycemia, steroid-induced hyperglycemia, and direct or indirect effects of SARS-CoV-2 on the pancreatic β-cells^58^. Overall, severe hyperglycemia is commonly observed in critically ill patients and is a recognized marker of disease severity^59^. In line, different reports have described that severe SARS-CoV-2 infection is associated with hyperglycemia in people with and without known diabetes^6,60^. One study from Wuhan of hospitalized, mainly elderly COVID-19 patients reported that 21.6% had a history of diabetes, and, based on the first glucose measurement upon admission, 20.8% were newly diagnosed with diabetes, and 28.4% were diagnosed with dysglycemia (fasting glucose 5.6–6.9 mmol/L and/or HbA_1c_ 5.7–6.4%)^6,58^. Importantly, the results from different studies emphasize that patients with newly diagnosed diabetes were more likely to be admitted to the intensive care unit, to require invasive mechanical ventilation due to ARDS, acute kidney injury, or shock, to have the longest hospital stays, and to display a significantly higher mortality rate^6,61–64^. Whereas reports on ACE2 expression in the pancreas together with a hypothesized SARS-CoV-2 mediated, direct pancreatic damage show somewhat conflicting results, stress and/or systemic treatment with corticosteroids are well-recognized risk factors for dysglycemia and diabetes also in patients affected by severe COVID-19^65–71^.

Besides preexisting metabolic disease, changes in lipoprotein composition and circulating lipid content, either due to altered intrahepatic processing or due to changes in cholesteryl ester transfer protein (CETP), phospholipid transfer protein (PLTP), and proprotein convertase subtilisin/kexin type 9 (PCSK9) activity in plasma, are a mainstay in patients with severe systemic inflammation. Together, both dyslipidemia and dysglycemia directly associate with the severity of acute pulmonary damage, and the high prevalence in early post-acute COVID-19 appears to reflect an ongoing resolution of inflammation.

This study has some limitations. First, we present an observational study, which is associated with typical limitations of that study design, including a lack of causal/mechanistic evaluation. Secondly, we observed a substantial patient drop-out throughout the prospective longitudinal follow-up until 360 days after COVID-19 onset. As expected, individuals with milder disease demonstrated a higher drop-out rate, as more patients from this subgroup fully recovered during the study, and thus were less motivated to attend late follow-up visits. This observation represents a potential study bias, especially for the late follow-up time points. Still, even at the last follow-up visit (360 days after disease onset), a representative patient cohort for the evaluation of patient outcomes related to the presence of dysglycemia or dyslipidemia was available for statistical analysis.

## Conclusions

In the prospective CovILD trial, metabolic disorders, including dysglycemia and dyslipidemia, were frequently found. Dysglycemia and dyslipidemia were related to a more severe course of acute COVID-19 and were associated with increased markers of thrombo-inflammation and a trend towards more severe structural lung impairment at early post-acute follow-up. Still, dysglycemia and dyslipidemia were not related to an impaired long-term pulmonary recovery from COVID-19.

## Supplementary Information

Supplementary Information.

## Data Availability

Relevant data is contained within the article and supplement. Additional data is available on request (contact: Thomas.Sonnweber@i-med.ac.at).
