# Supplementary material for: Pulmonary recovery from COVID-19 in patients with metabolic diseases: a longitudinal prospective cohort study
Source: Sci Rep. 2023 Feb 14;13:2599. doi: 10.1038/s41598-023-29654-1 (PMC9926446; doi:10.1038/s41598-023-29654-1)
Supplement: Supplementary file 1 — Supplementary Information. [file 41598_2023_29654_MOESM1_ESM.docx]

**Supplementary Material**

**Supplementary Methods**

**Statistical analysis**

The study data set was handled and analyzed with GraphPad Prism software version 8.0 (GraphPad Software, San Diego, CA, USA), statistical analysis software package (IBM SPSS Statistics version 27.0; IBM, Armonk, NY, USA) and R version 4.2.0R version 4.2.0. Handling and transformation of tabular data was accomplished by the packages *tidyverse* (1), *rlang* (2) and the development package *trafo* (<https://github.com/PiotrTymoszuk/trafo>). Text data were handled (search, extraction, replacement) with *stringi* (3). For descriptive statistic, correlation analysis and statistical hypothesis testing, the packages *rstatix* (4) and *ExDA* (<https://github.com/PiotrTymoszuk/ExDA>) were employed. Ordinal logistic modeling and model diagnostic was done with *MASS* (5), *broom* (6), *caret* (7) and the development packages *lmqc* (<https://github.com/PiotrTymoszuk/lmqc>) and *caretExtra* (<https://github.com/PiotrTymoszuk/caretExtra>).

Results were visualized with the packages *ggplot* (8), *ExDA* and *cowplot* (9). Tables were created with *flextable* (10). Parts of the manuscript and supplementary material were written in the *rmarkdown* environment (11). Tables and figures were handled (insertion and reference) with the development package *figur* (<https://github.com/PiotrTymoszuk/figur>). Text parts were rendered as Word and PDF documents with *bookdown* (12) and *knitr* (13).

##

## Variable transformation

Participants were dichotomized according to the age cutoff of 60 years. Overweight was defined as body mass index (BMI) > 25 $kg/m^{2}$, obesity as BMI > 30 $kg/m^{2}$. Dyslipidemia was defined with triglycerides (TG) > 150 mg/dL or low high density lipoprotein (HDL, < 40 mg/dL for males or HDL < 50 mg/dL females). Dysglycemia was defined as HbA1c ≥ 5.7%. Participants were classified according to acute COVID-19 severity as mild (WHO ordinal scale for clinical improvement: 1 - 2), moderate (WHO: 3 - 4) and severe (WHO: $\geq$ 5). Elevated C-reactive protein (CRP, > 0.5 mg/dL), interleukin 6 (IL6, > 7 pg/mL) and TG (> 150 mg/dL) were defined with standard cutoffs. Chest CT abnormalities were classified as none (CT severity score [CTSS]: 0), mild (CTSS: 1 - 5), moderate (CTSS: 6 - 10) and severe (CTSS: $\geq$ 11) (14). Some variables were in addition transformed with the log function to improve normality and homogeneity of variances.

##

## Explorative data analysis, normality and variance homogeneity testing

Descriptive statistic of the study variables and differences between the COVID-19 severity strata were investigated with the *ExDA* and *rstatix* (4) packages. Differences in categorical variables between the COVID-19 severity strata were assessed by $\chi^{2}$ test with Cramer V effect size statistic. Differences in numeric variables between the COVID-19 severity strata were investigated by Kruskal-Wallis test with $\eta^{2}$ effect size statistic (data not shown).

Normality of numeric variable distribution in groups defined by age class and COVID-19 severity or dyslipidemia/dysglycemia status and follow-up was assessed with Shapiro-Wilk test. Homogeneity of variances in these strata was investigated by Levene test. Distribution testing was performed with *ExDA* and *rstatix* (4). D-dimer (DDimer), ferritin (FT), HDL, adiponectin (ADIPOQ) and leptin (LEP) concentrations were log-transformed to improve normality before modeling. IL6, CRP and TG were dichotomized with the cutoffs described above to meet the ordinal logistic regression assumptions.

##

## Correlation and time course analysis

Pair-wise association of non-transformed levels of metabolic (TG, HDL, ADIPOQ, LEP) and inflammatory biomarkers (CRP, IL6, DDimer, FT) was analyzed by Spearman’s correlation (package *ExDA*).

Changes in non-transformed levels of inflammatory and metabolic biomarkers between consecutive follow-ups in participant strata defined by dyslipidemia and dysglycemia status were investigated by Friedman test with Kendall’s W effect size statistic (*rstatix*) (4). Differences between the strata at specific follow-ups were assessed by Holm-corrected Mann-Whitney U post-hoc test (*ExDA*).

##

## Ordinal logistic modeling

Classes of chest CT abnormality were defined as described above. Effects of inflammatory biomarkers (CRP, IL6, DDimer, FT), metabolic parameters (TG, HDL, ADIPOQ, LEP) and metabolic disorders (obesity, dyslipidemia, dysglycemia) on the severity of chest CT abnormalities at the 60-day follow-up were investigated by ordinal logistic modeling (package *MASS*) (5). Three types of models were constructed for each explanatory variable: (1) uni-variable models, (2) models including age class and sex as confounders, and (3) models including age class, sex and acute COVID-19 severity as confounders (package *lmqc*). The confounders selected for analysis are canonical risk factors of persistent chest CT abnormality (**Supplementary** **Figure 3, Supplementary** **Table 1 and 2**). Normality and homogeneity of variance of the model residuals were assessed by Shapiro-Wilk and Levene tests, respectively (packages *lmqc* and *broom*) (6). The proportionality of odds was gauged by Brant test (package *brant*) (15,16). Metrics of classification performance of the models (classification error, Cohen’s $\kappa$ and confusion matrix) were computed with the *caret* package tools (7) (**Supplementary** **Table 3**). Finally, model coefficient estimates expressed as odds ratios (OR) and their confidence intervals were extracted. OR significance (OR $\neq$ 1) was determined by a two-tailed T-test (**Supplementary** **Table 4**).

Multi-parameter modeling of chest CT abnormality severity was done by ordinal logistic regression with Akaike information criterion (AIC) driven backwards elimination of non-significant terms as described by us recently in the CovILD study data set (14). Briefly, full models including age class, sex, acute COVID-19 severity, CRP, IL6, DDimer, FT, TG, HDL, ADIPOQ, LEP, obesity, dyslipidemia and dysglycemia at the 60-day follow-up were constructed. The full models were subsequently optimized by backwards elimination (function stepAIC(), package *MASS*) (5). Assumptions of the model residuals distributions, classification performance and inference were analyzed as described above. Reproducibility and proper parameterization of the models was investigated with cross-validation (10-fold, 50 repeats, packages *caret* and *caretExtra*) (7). As demonstrated in **Supplementary** **Figure 4**, comparable classification errors and $\kappa$ accuracy statistics of the models in the training data set and cross-validation suggest that none of the multi-parameter models suffered from overfitting.

##

## Data and code availability

The anonymized study data set will be provided on request to the corresponding author. The R analysis pipeline is available at <https://github.com/PiotrTymoszuk/covild_metabolic>.

**Supplementary Results**

# Supplementary Tables

Supplementary Table 1. Classification error and accuracy of logistic ordinal modeling of chest CT abnormality severity at the day 60 follow-up as a function of age, sex and COVID-19 severity.

| **Variable** | **Classification statistics** |
| --- | --- |
| age | class. error = 0.56 κ = 0.22 |
| sex | class. error = 0.54 κ = 0.24 |
| COVID-19 severity | class. error = 0.44 κ = 0.37 |

**Supplementary Table 2:** Classification error and accuracy of logistic ordinal modeling of chest CT abnormality severity at the day 60 follow-up as a function of inflammatory and metabolic parameters.

| **Variable** | **Unadjusted univariable model** | **Age/sex-adjusted model** | **Age/sex/severity-adjusted model** |
| --- | --- | --- | --- |
| CRP, 60d FUP | class. error = 0.67 κ = 0 | class. error = 0.5 κ = 0.31 | class. error = 0.44 κ = 0.37 |
| IL6, 60d FUP | class. error = 0.67 κ = 0 | class. error = 0.46 κ = 0.37 | class. error = 0.43 κ = 0.39 |
| log DDimer, 60d FUP | class. error = 0.62 κ = 0.13 | class. error = 0.46 κ = 0.36 | class. error = 0.43 κ = 0.4 |
| log FT, 60d FUP | class. error = 0.58 κ = 0.17 | class. error = 0.51 κ = 0.29 | class. error = 0.44 κ = 0.37 |
| TG, 60d FUP | class. error = 0.66 κ = 0.09 | class. error = 0.5 κ = 0.3 | class. error = 0.44 κ = 0.37 |
| log HDL, 60d FUP | class. error = 0.61 κ = 0.12 | class. error = 0.48 κ = 0.34 | class. error = 0.44 κ = 0.37 |
| log ADIPOQ, 60d FUP | class. error = 0.7 κ = -0.033 | class. error = 0.49 κ = 0.33 | class. error = 0.44 κ = 0.37 |
| log LEP, 60d FUP | class. error = 0.63 κ = 0.086 | class. error = 0.47 κ = 0.35 | class. error = 0.44 κ = 0.38 |
| obesity^a^ | class. error = 0.67 κ = 0 | class. error = 0.46 κ = 0.36 | class. error = 0.43 κ = 0.4 |
| dyslipidemia, 60d FUP^b^ | class. error = 0.67 κ = 0 | class. error = 0.49 κ = 0.33 | class. error = 0.44 κ = 0.37 |
| dysglycemia, 60d FUP^c^ | class. error = 0.67 κ = 0 | class. error = 0.48 κ = 0.33 | class. error = 0.44 κ = 0.37 |
| ^a^body mass index (BMI) > 30 kg/m² | | | |
| ^b^TG > 150 mg/dL or HDL < 40 mg/dL (male) or HDL < 50 mg/dL (female) | | | |
| ^c^HbA1c ≥ 5.7% | | | |

Supplementary Table 3: Logistic ordinal modeling of chest CT abnormality severity at the day 60 follow-up as a function of age, sex and COVID-19 severity.

Odds ratios (OR) with 95% confidence intervals, p values, numbers of cases in the strata and the total observation counts are presented.

| **Variable** | **Strata** | **OR** |
| --- | --- | --- |
| age, years | >60 | OR = 5.1 [2.7 - 9.92] p = 1.1e-06 strata: n = 55 complete: n = 145 |
| sex | male | OR = 5.7 [3 - 112] p = 1.8e-07 strata: n = 82 complete: n = 145 |
| COVID-19 severity | moderate | OR = 17 [5.8 - 532] p = 6.2e-07 strata: n = 38 complete: n = 145 |
|  | severe | OR = 74 [26 - 2402] p = 2.5e-14 strata: n = 73 complete: n = 145 |

Supplementary Table 4. Logistic ordinal modeling of chest CT abnormality severity at the day 60 follow-up as a function of inflammatory and metabolic parameters.

| **Variable** | **Strata** | **Unadjusted OR** | **Age/sex-adjusted OR** | **Age/sex/severity-adjusted OR** |
| --- | --- | --- | --- | --- |
| CRP, 60d FUP | >0.5 mg/dL | OR = 3 [1.3 - 7.32] p = 0.014 strata: n = 23 complete: n = 145 | OR = 3.8 [1.5 - 102] p = 0.008 strata: n = 23 complete: n = 145 | OR = 2.9 [1.1 - 8.22] p = 0.034 strata: n = 23 complete: n = 145 |
| IL6, 60d FUP | >7 pg/mL | OR = 9.8 [3 - 452] p = 0.00067 strata: n = 15 complete: n = 145 | OR = 9.3 [2.5 - 472] p = 0.0024 strata: n = 15 complete: n = 145 | OR = 7.2 [1.9 - 382] p = 0.0085 strata: n = 15 complete: n = 145 |
| log DDimer, 60d FUP, pg/mL |  | OR = 2.2 [1.5 - 3.52] p = 0.00021 complete: n = 145 | OR = 1.6 [0.97 - 2.52] ns (p = 0.071) complete: n = 145 | OR = 1.8 [1.1 - 32] p = 0.024 complete: n = 145 |
| log FT, 60d FUP, ng/mL |  | OR = 2.1 [1.5 - 32] p = 5.8e-05 complete: n = 145 | OR = 1.7 [1.2 - 2.62] p = 0.0077 complete: n = 145 | OR = 1.3 [0.84 - 22] ns (p = 0.24) complete: n = 145 |
| TG, 60d FUP | >150 mg/dL | OR = 1.9 [1 - 3.72] p = 0.042 strata: n = 44 complete: n = 145 | OR = 1.6 [0.78 - 3.42] ns (p = 0.19) strata: n = 44 complete: n = 145 | OR = 0.96 [0.45 - 2.12] ns (p = 0.92) strata: n = 44 complete: n = 145 |
| log HDL, 60d FUP, mg/dL |  | OR = 0.26 [0.093 - 0.72] p = 0.008 complete: n = 145 | OR = 0.82 [0.25 - 2.72] ns (p = 0.74) complete: n = 145 | OR = 1.3 [0.38 - 4.62] ns (p = 0.66) complete: n = 145 |
| log ADIPOQ, 60d FUP, ng/mL |  | OR = 0.74 [0.43 - 1.32] ns (p = 0.28) complete: n = 145 | OR = 0.94 [0.49 - 1.82] ns (p = 0.85) complete: n = 145 | OR = 1.1 [0.56 - 2.12] ns (p = 0.83) complete: n = 145 |
| log LEP, 60d FUP, ng/mL |  | OR = 0.56 [0.37 - 0.822] p = 0.0034 complete: n = 145 | OR = 0.85 [0.54 - 1.32] ns (p = 0.46) complete: n = 145 | OR = 0.72 [0.44 - 1.22] ns (p = 0.17) complete: n = 145 |
| obesity^a^ |  | OR = 0.9 [0.43 - 1.92] ns (p = 0.79) strata: n = 27 complete: n = 145 | OR = 0.62 [0.27 - 1.42] ns (p = 0.26) strata: n = 27 complete: n = 145 | OR = 0.42 [0.17 - 12] ns (p = 0.056) strata: n = 27 complete: n = 145 |
| dyslipidemia, 60d FUP^b^ |  | OR = 1.2 [0.6 - 2.62] ns (p = 0.56) strata: n = 32 complete: n = 145 | OR = 0.91 [0.39 - 2.12] ns (p = 0.83) strata: n = 32 complete: n = 145 | OR = 0.62 [0.26 - 1.52] ns (p = 0.29) strata: n = 32 complete: n = 145 |
| dysglycemia, 60d FUP^c^ |  | OR = 1.5 [0.8 - 2.62] ns (p = 0.22) strata: n = 62 complete: n = 145 | OR = 0.87 [0.44 - 1.72] ns (p = 0.67) strata: n = 62 complete: n = 145 | OR = 0.91 [0.44 - 1.92] ns (p = 0.8) strata: n = 62 complete: n = 145 |
| ^a^body mass index (BMI) > 30 kg/m² | | | | |
| ^b^TG > 150 mg/dL or HDL > 40 mg/dL (male) or HDL > 50 mg/dL (female) | | | | |
| ^c^HbA1c ≥ 5.7% | | | | |

Odds ratios (OR) with 95% confidence intervals, p values, numbers of cases in the strata and the total observation counts are presented for univariable/unadjusted models, age/sex- and age/sex/severity-adjusted models.

# Supplementary Figures


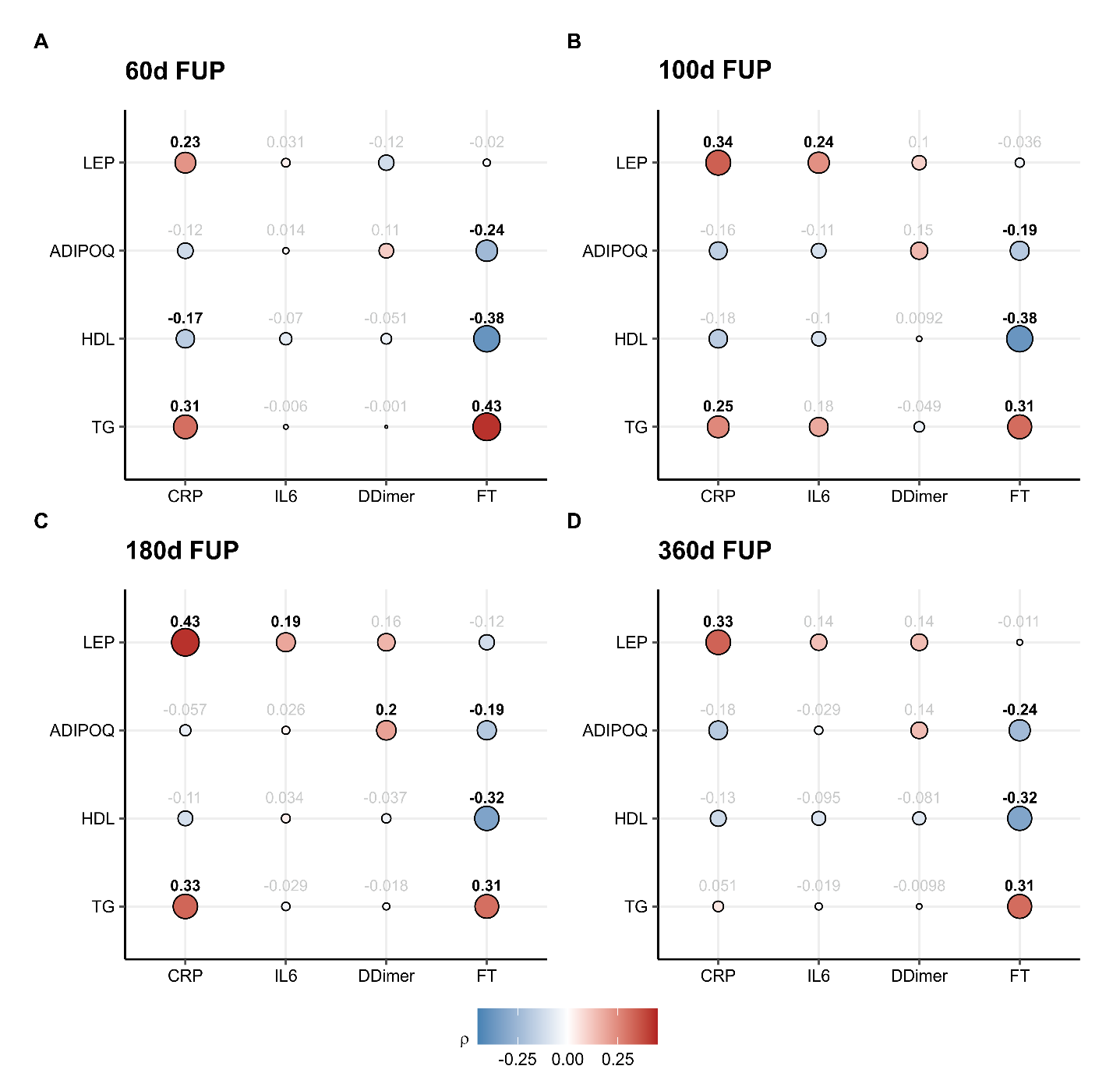


**Supplementary Figure 1. Correlation of metabolic and inflammatory parameters at the 60-, 100-, 180- and 360-day follow-up.** Correlation of inflammatory (C-reactive protein [CRP], interleukin 6 [IL6], D-dimer [DDimer], ferritin [FT]), metabolic bio-markers (triglycerides [TG], high-density lipoprotein [HDL], adiponectin [ADIPOQ], leptin [LEP]) at the 60 (A), 100 (B), 180 (C) and 360-day follow-up (D) was investigated by Spearman test. Correlation coefficient ($\rho$) are presented in bubble plots. Point size represents the absolute value of $\rho$, point colour codes for the $\rho$ value. Points are labeled with the $\rho$ values. The values for significant correlations are highlighted in bold. N_60days_=145, N_100days_ =138, N_180days_ =119, N_360days_ =92.


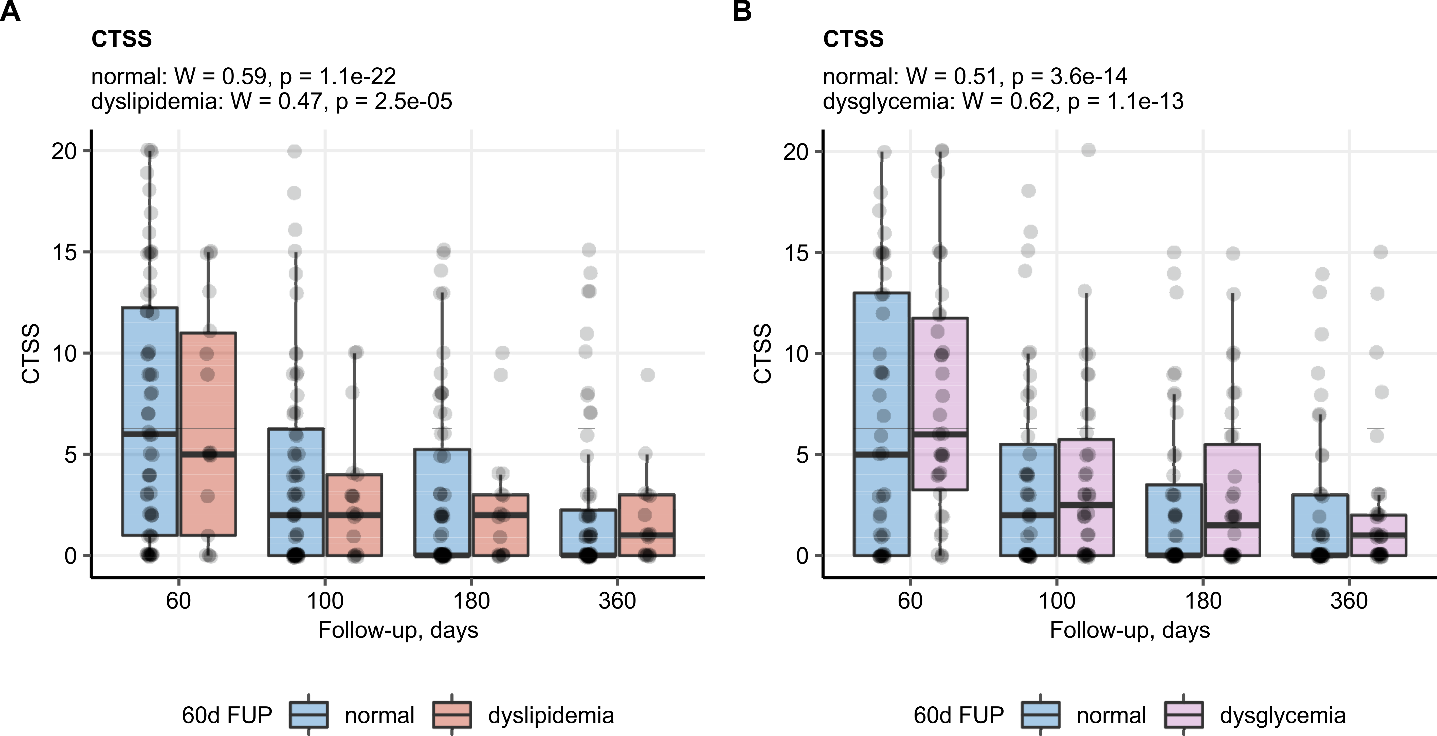


**Supplementary Figure 2. Time course of chest CT severity score at the 60-, 100-, 180- and 360-day follow-up in study participants with normal metabolic profile, dyslipidemia and dysglycemia at the 60-day visit.** Chest CT abnormality severity was assessed with CT severity score (CTSS) at the consecutive follow-ups in participants with normal lipid profile and dysglycemia (A) and in participants with normal glycemia and dysglycemia (B). The significance of changes in the concentrations in time was determined for each of the strata by Friedman test with Kendall’s W effect size statistic. Differences between the strata at specific follow-ups were assessed by Holm-corrected Mann-Whitney post-hoc test. Median concentrations with interquartile ranges (IQR) are presented in box plots, whiskers span over 150% IQR. Points represent single observations. Effect size statistic and Friedman test p values are displayed in the plot captions. Significant results of the post-hoc test are shown in the plots. N_total_=145, N_dysglycemia_=62, N_dyslipidemia_ =32.

**
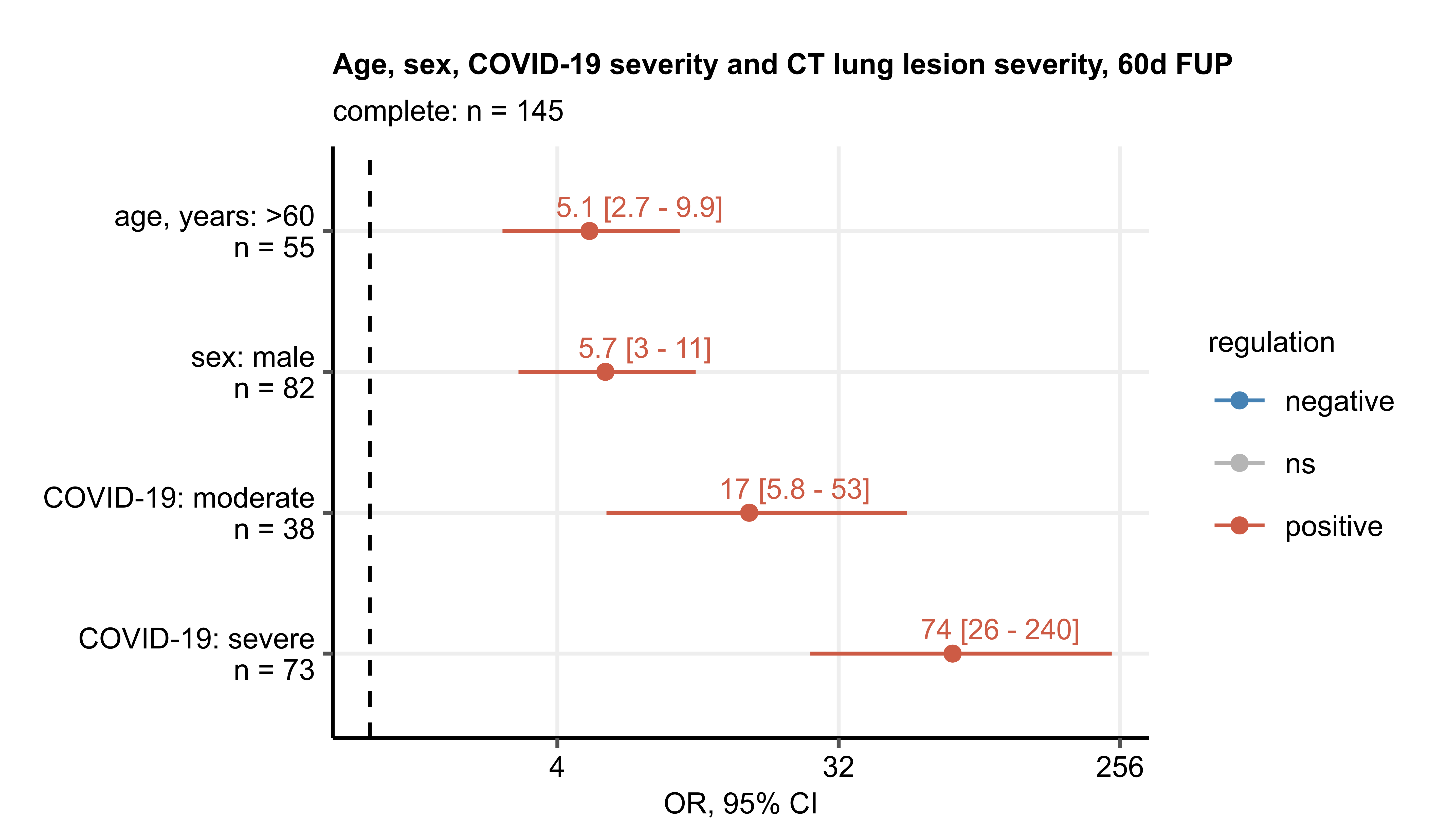
**

**Supplementary Figure 3. Logistic ordinal modeling of chest CT abnormality severity at the 60-day follow-up as a function of age, sex and COVID-19 severity.**

Chest CT abnormalities were classified as none (CT severity score [CTSS]: 0), mild (CTSS: 1 - 5), moderate (CTSS: 6 - 10) and severe (CTSS: $\geq$ 11). Effects of participant age class (below/above 60 years), sex and acute COVID-19 severity (mild/moderate/severe) on chest CT abnormality severity at the 60-day follow-up were assessed by univariable ordinal logistic regression. Odds ratio (OR) with 95% confidence intervals (CI) are shown in a Forest plot. Point colour codes for significance and model estimate signs. Points are labeled with OR and 95% CI values.

**
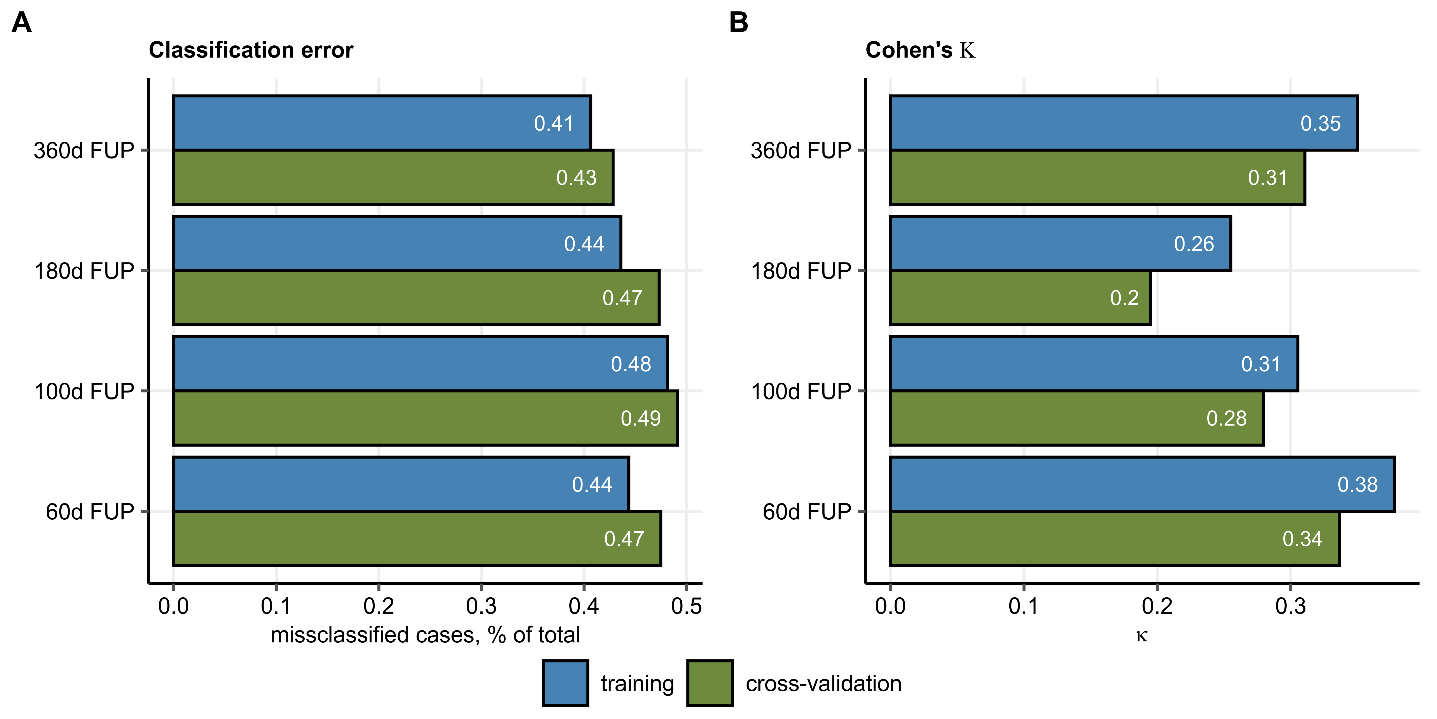
**

**Supplementary Figure 4. Accuracy of chest CT abnormality severity prediction by multi-parameter ordinal logistic modeling at 60-, 100-, 180- and 360-day follow-up.** Chest CT abnormalities were classified as none (CT severity score [CTSS]: 0), mild (CTSS: 1 - 5), moderate (CTSS: 6 - 10) and severe (CTSS: $\geq$ 11). Additive effects of age class (below/above 60 years), sex, acute COVID-19 severity (mild., moderate, severe), inflammatory parameters (C-reactive protein [CRP], interleukin 6 [IL6], D-dimer [DDimer], ferritin [FT]), metabolic bio-markers (triglycerides [TG], high-density lipoprotein [HDL], adiponectin [ADIPOQ], leptin [LEP]) and metabolic disorders (obesity, dyslipidemia and dysglycemia) recorded at the 60-day follow-up on chest CT abnormality severity was modeled by ordinal logistic regression with Akaike information criterion (AIC) driven backwards elimination of non-significant terms. Classification error (A) and accuracy of CT abnormality strata assignment expressed by Cohen’s $\kappa$ statistic (B) are presented for the models’ training data sets and cross-validation (10-fold, 50-repeats).

**
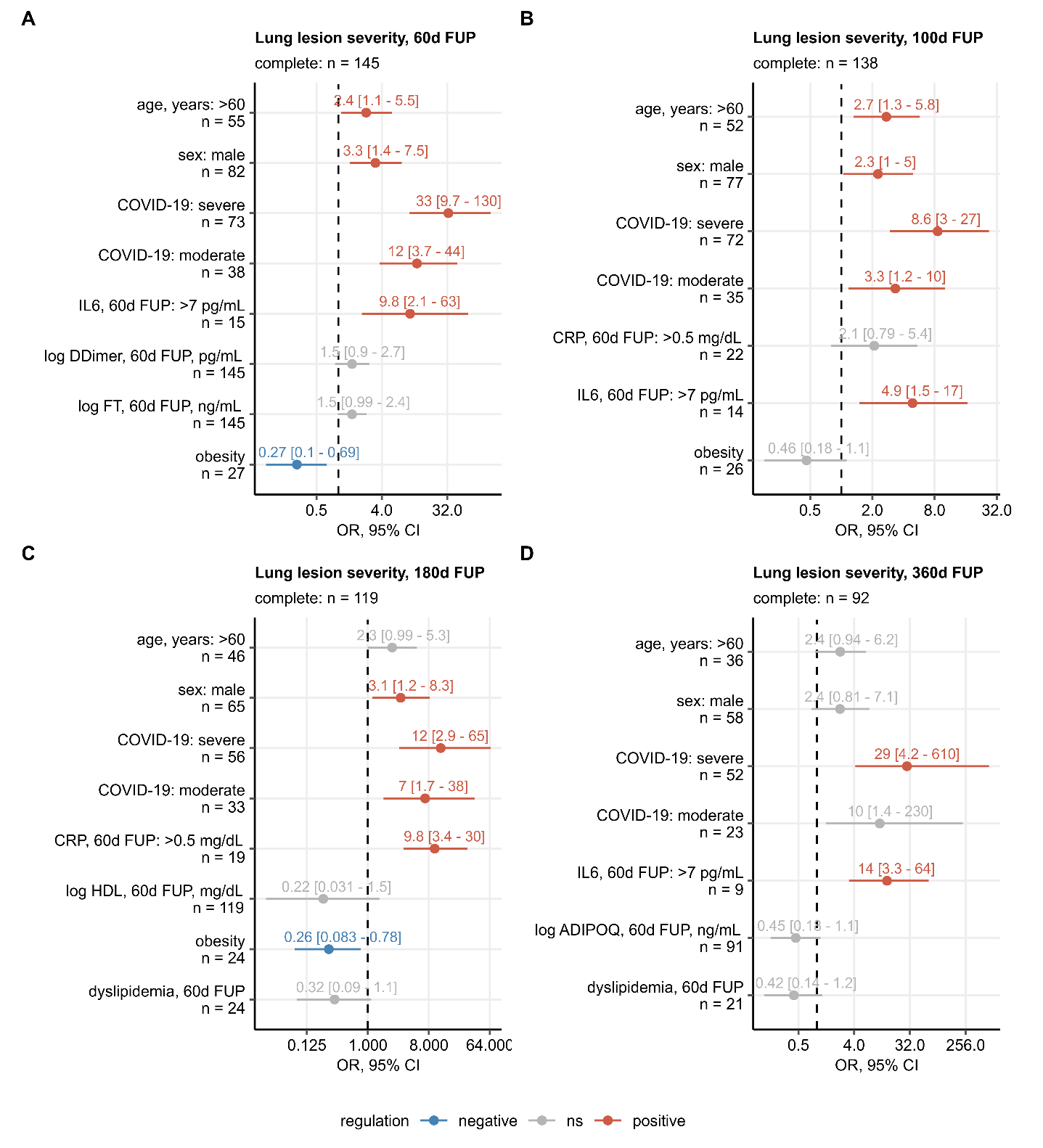
**

**Supplementary Figure 5. Results of multi-parameter logistic ordinal modeling of CT lung abnormality severity at the 60-, 100-, 180- and 360-day follow-up as a function of age, sex, COVID-19 severity, and inflammatory and metabolic parameters.** Chest CT abnormalities were classified as none (CT severity score [CTSS]: 0), mild (CTSS: 1 - 5), moderate (CTSS: 6 - 10) and severe (CTSS: $\geq$ 11). Additive effects of age class (below/above 60 years), sex, acute COVID-19 severity (mild., moderate, severe), inflammatory parameters (C-reactive protein [CRP], interleukin 6 [IL6], D-dimer [DDimer], ferritin [FT]), metabolic bio-markers (triglycerides [TG], high-density lipoprotein [HDL], adiponectin [ADIPOQ], leptin [LEP]) and metabolic disorders (obesity, dyslipidemia and dysglycemia) recorded at the 60-day follow-up on chest CT abnormality severity was modeled by ordinal logistic regression with Akaike information criterion (AIC) driven backwards elimination of non-significant terms. Odds ratio (OR) with 95% confidence intervals (CI) for the explanatory variables of the final multi-parameter models are shown in Forest plots. Point colour codes for significance and model estimate signs. Points are labeled with OR and 95% CI values.

# Supplementary References

1. Wickham H, Averick M, Bryan J, Chang W, McGowan L, François R, Grolemund G, Hayes A, Henry L, Hester J, et al. Welcome to the Tidyverse. *Journal of Open Source Software* (2019) 4:1686. doi: [10.21105/joss.01686](https://doi.org/10.21105/joss.01686)

2. Henry L, Wickham Hadley. rlang: Functions for Base Types and Core R and ’Tidyverse’ Features. (2022) <https://cran.r-project.org/web/packages/rlang/index.html>

3. Gagolewski M, Tartanus B. CRAN - Package stringi. (2021) <https://cran.r-project.org/web/packages/stringi/index.html>

4. Kassambara A. rstatix: Pipe-Friendly Framework for Basic Statistical Tests. (2021) <https://cran.r-project.org/package=rstatix>

5. Ripley B. MASS: Support Functions and Datasets for Venables and Ripley’s MASS. (2022) <https://cran.r-project.org/package=MASS>

6. Robinson D. broom: An R Package for Converting Statistical Analysis Objects Into Tidy Data Frames. (2014) <http://arxiv.org/abs/1412.3565>

7. Kuhn M. Building predictive models in R using the caret package. *Journal of Statistical Software* (2008) 28:1–26. doi: [10.18637/jss.v028.i05](https://doi.org/10.18637/jss.v028.i05)

8. Wickham Hadley. *ggplot2: Elegant Graphics for Data Analysis*. 1st ed. New York: Springer-Verlag (2016). <https://ggplot2.tidyverse.org>

9. Wilke CO. *Fundamentals of Data Visualization: A Primer on Making Informative and Compelling Figures*. 1st ed. Sebastopol: O’Reilly Media (2019).

10. Gohel D. flextable: Functions for Tabular Reporting. (2022) <https://cran.r-project.org/web/packages/flextable/index.html>

11. Allaire J, Xie Y, McPherson J, Luraschi J, Ushey K, Atkins A, Wickham H, Cheng J. rmarkdown: Dynamic Documents for R. (2022) <https://cran.r-project.org/web/packages/rmarkdown/index.html>

12. Xie Y. *Bookdown : authoring books and technical documents with R Markdown*. (2016).

13. Xie Y. knitr: A General-Purpose Package for Dynamic Report Generation in R. (2022) <https://cran.r-project.org/web/packages/knitr/index.html>

14. Luger AK, Sonnweber T, Gruber L, Schwabl C, Cima K, Tymoszuk P, Gerstner AK, Pizzini A, Sahanic S, Boehm A, et al. Chest CT of Lung Injury 1 Year after COVID-19 Pneumonia: The CovILD Study. *Radiology* (2022) doi: [10.1148/RADIOL.211670](https://doi.org/10.1148/RADIOL.211670)

15. Brant R. Assessing Proportionality in the Proportional Odds Model for Ordinal Logistic Regression. *Biometrics* (1990) 46:1171. doi: [10.2307/2532457](https://doi.org/10.2307/2532457)

16. Schlegel B, Steenbergen M. brant: Test for Parallel Regression Assumption. (2022) <https://cran.r-project.org/web/packages/brant/index.html>
